# The Relationship Between Sociodemographics and Environmental Values Across Seven European Countries

**DOI:** 10.3389/fpsyg.2020.02253

**Published:** 2020-08-28

**Authors:** Rebecca J. Sargisson, Judith I. M. De Groot, Linda Steg

**Affiliations:** ^1^Department of Psychology, Faculty of Behavioural and Social Sciences, University of Groningen, Groningen, Netherlands; ^2^School of Psychology, Division of Arts, Law, Psychology, and Social Sciences, University of Waikato, Tauranga, New Zealand; ^3^Department of Marketing, Faculty of Economics and Business, University of Groningen, Groningen, Netherlands

**Keywords:** environmental, segmentation, sociodemographics, gender, green consumers, political orientation, values

## Abstract

Given the importance of environmental values (altruistic, biospheric, and egoistic) to pro-environmental behavior, it would be useful to segment the population – an approach known as market segmentation – to tailor pro-environmental messages more effectively. Sociodemographic variables are popular targets for segmentation, as such variables are often knowable in the absence of more nuanced information about individuals. However, evidence for the relationship between sociodemographics and environmental values is sparse, and contradictory. We examined the extent to which popular sociodemographic variables (gender, age, income, education, urbanization level, and political orientation) were predictive of environmental values for 11,820 participants across seven European countries. Overall, sociodemographics were hardly related to environmental values. Only gender and political orientation were weakly but significantly related to environmental values, whereby men and right-wingers showed weaker altruistic and biospheric, and stronger egoistic, values than women and left-wingers. We conclude that sociodemographic variables cannot be considered a suitable proxy for environmental values, and thus that behavior-change campaigns might be more impactful when focused on alternative segmentation strategies in relation to environmental aims.

## Introduction

Information campaigns to change behavior are more successful when their messages are tailored to specific groups of people (Steg, 2008), a concept known in economic circles as market segmentation (do Paço et al., 2009). With the increasing acknowledgment that human behaviors need to change to mitigate climate change, companies and governments need to encourage pro-environmental behaviors (Diamantopoulos et al., 2003; Jain and Kaur, 2006), which are behaviors that benefit the environment, or harm it as little as possible (Steg and Vlek, 2009). There is evidence that environmental values, specifically altruistic, biospheric, and egoistic values, are important determinants of pro-environmental behavior (Stern et al., 1995; Karp, 1996; Nordlund and Garvill, 2002). In this context, market segmentation helps companies and governmental bodies to more effectively target consumers and people with specific environmental values (Jain and Kaur, 2006), as people differ in the extent to which they endorse altruistic, biospheric, and egoistic values in their lives (Deng et al., 2006).

Market segmentation has received considerable attention (for a review see, e.g., Diamantopoulos et al., 2003). Although more sophisticated segmentation approaches are possible and promising (e.g., Poortinga and Darnton, 2016), using sociodemographics alone, such as gender, age, household type and income, has been popular for segmenting and profiling populations due to their ease of measurement and application (Myers, 1996). Indeed, many companies focus primarily on sociodemographic variables in their market segmentation strategies (McDonald and Dunbar, 1998). However, empirical support of the importance of sociodemographics in relation to environmental values seems to be limited and ambiguous at best (Van Liere and Dunlap, 1980). Gaining more certainty about the extent to which sociodemographics are relevant in relation to environmentally relevant values is important for marketers and policy makers alike, so they can adapt their segmentation strategies accordingly. We aimed to add to the existing literature on this topic using data from seven European countries. We explored the strength of the relationship between the sociodemographic variables of gender, age, social class, urbanization level of residence, and political orientation and environmental values.

Schwartz (1994) described values, such as freedom, equality, and protecting the environment, as principles that guide a person or social entity to achieve desirable goals. One of the two value dimensions described by Schwartz (1992) is the self-enhancement vs. self-transcendent dimension. The dimension reflects the degree to which a person values the welfare and interests of others (self-transcendent) as opposed their own personal interests (self-enhancement). Values include beliefs about the desirability of certain end-states and, because they are abstract in nature, they allow for predictions across a broad range of contexts and behaviors, including a variety of pro-environmental attitudes and behaviors (Seligman and Katz, 1996).

Three types of values are proposed to be particularly relevant in the environmental domain (Stern et al., 1993; Steg et al., 2005; De Groot and Steg, 2009). Altruistic values motivate people to maximize outcomes for the welfare of other human beings. Biospheric values lead to a focus on the interests of non-human species and the biosphere. Egoistic values involve the goal of maximizing outcomes for the self, such as personal wealth or power (De Groot and Steg, 2007, 2008).

Behaving pro-environmentally often produces immediate negative consequences for the individual (e.g., reduction in comfort, higher financial costs), but positive long-term consequences for society and the environment. In line with this assumption, research shows that altruistic and biospheric values are positively related to pro-environmental attitudes, intentions, and behaviors (Marshall et al., 2019; Shi et al., 2019), while egoistic values are often negatively related to them (Honkanen and Verplanken, 2004; Nilsson et al., 2004; De Groot and Steg, 2008, 2010; Steg et al., 2011; Bouman et al., 2018). As altruistic, biospheric, and egoistic values are all relevant in relation to pro-environmental behavior, we refer to these three values as “environmental values” throughout this paper.

Although less research has examined the relationship between sociodemographics and environmental values, the relationship between sociodemographics and environmental concern has received more extensive focus (see Diamantopoulos et al., 2003, for a review). Steg et al. (2011) defined environmental concern as the “evaluation of the seriousness of environmental problems” (p. 351), and research suggests that sociodemographic factors influence environmental concern (Fransson and Gärling, 1999; Poortinga et al., 2019). However, environmental values might be more appropriate for segmentation strategies than environmental concern because values describe broader, overarching goals that remain stable across an adult’s life (Stern and Dietz, 1994), whereas environmental concern has been seen to change rapidly in a population over relatively short periods (Dunlap and Scarce, 1991). Additionally, studying the relationships between sociodemographics and each of the three environmental values separately might suggest ways to target behavior-change campaigns to emphasize either their altruistic, biospheric, or egoistic attributes, rather than focusing simply on environmental concern.

However, there appears to be little research on whether sociodemographic factors are associated with environmental values. It is reasonable to expect that sociodemographic variables will be similarly related to environmental values, because environmental concern is positively related to biospheric values (Steg et al., 2011) and also related, albeit less strongly, to altruistic and egoistic values (Steg et al., 2011), however the strength of these relationships might be too weak to use effectively in market segmentation strategies. We used large, representative samples from seven European countries [France, Greece, Hungary, the Netherlands, Norway, Switzerland, and the United Kingdom (UK)] to examine whether relationships between environmental values and sociodemographic variables are similar across populations.

Van Liere and Dunlap (1980) proposed a framework four decades ago including five popular hypotheses about the relationships between the sociodemographics of gender, age, social class, urbanization level, and political orientation and environmental concern. It comes as no surprise that years after the introduction of these beliefs, these five sociodemographics are still regarded as important segmentation and profiling factors in relation to targeting environmentally conscious people (Diamantopoulos et al., 2003; Jain and Kaur, 2006). Below, we will reflect on these five beliefs in relation to empirical evidence with respect to environmental concern, and, we will translate evidence to propose what these beliefs imply in relation to environmental values (i.e., altruistic, biospheric, and egoistic values).

In regards to the relationship between gender and environmental concern, Van Liere and Dunlap (1980) described two conflicting hypotheses. On one hand, men are assumed by some researchers to be *more* concerned about environmental problems than women, due to their greater involvement in politics and community issues and their higher educational levels. On the other hand, other researchers argue that men may be *less* concerned about the environment than women because of their greater concern with economic issues (Van Liere and Dunlap, 1980).

According to Van Liere and Dunlap (1980), evidence for the gender hypothesis was “meager” and “inconclusive” (p. 191) at the time their article was published, and they concluded, based on the limited empirical evidence they had, that there was no substantial association between gender and environmental concern.

However, more recent research has shown that women are more concerned about the environment (e.g., Van Liere and Dunlap, 1981; Mohai, 1992; Blocker and Eckberg, 1997; Casey and Scott, 2006; Dutcher et al., 2007; Hirsh, 2010; Swami et al., 2010; Burn et al., 2012; Huddart-Kennedy et al., 2015; Xiao and McCright, 2015; Echavarren, 2017; Mueller and Mullenbach, 2018; *r*s = −0.02 to 0.21), but the relationship is often weak (Casey and Scott, 2006; Dutcher et al., 2007, β = 0.11; Hirsh, 2010,β = −0.07; Mohai, 1992, β = 0.10; Nord et al., 1998, β = −0.04, β = −0.05; Swami et al., 2010, β = −0.14 and −0.22; Xiao and McCright, 2015, β = 0.04). In a recent 36-country investigation, significant gender differences in environmental concern were found for only seven countries (Lewis et al., 2019). Where the gender difference was the most extreme (English-speaking Western democracies), women were still only 7% more likely than men to call climate change a serious problem, while the difference was halved for the rest of Europe and non-existent in the rest of the world (Lewis et al., 2019). Relatedly, Chan et al. (2019) noted that the pattern of gender differences in environmental concern is less clear in studies conducted outside the United States. Li and Chen (2018), for example, showed that the Chinese women in their sample reported *less* environmental concern than the men (*b* = −0.46). Consistent with these mixed findings, Chan et al. (2019) argue that more cross-country validation is needed to validate the gender hypothesis.

Translating the findings on the relationship between gender and environmental concern to environmental values, researchers have argued that women should have stronger altruistic values because they have higher levels of empathy and a lower social dominance orientation; defined as the extent to which people support a hierarchical social system (Milfont and Sibley, 2016; Graça et al., 2018), likely due to socialization of gender roles. In support of this argument, women report stronger levels of social responsibility (Zeleny et al., 2000) and are more compassionate than men (Beutel and Marini, 1995). At least one study has found that women have stronger altruistic values (i.e., Ndofirepi, 2019) than men, but the effect size was relatively small (*d* = 0.22). Using Schwartz’s original 10-value scale, a meta-analytic study of 127 cultural groups showed that women attributed more importance to self-transcendent values than men (Schwartz and Rubel, 2005), but the effect size was small (*d* = 0.21 to 0.29).

In relation to biospheric values, one argument is that women’s disadvantaged position in society should produce a greater perceived vulnerability to environmental risks (Flynn et al., 1994), and hence, that women should have stronger biospheric values than men (Milfont and Sibley, 2016). However, biospheric values were not shown to differ significantly by gender in at least one study (i.e., Ndofirepi, 2019; *d* = 0.10).

With regard to egoistic values, because it has been shown that men are more individualistic than women (Grendstad and Sundback, 2003), it is assumed that men will more strongly endorse egoistic values than women will. The sparse empirical evidence related to this hypothesis, however, seems to show no difference in egoistic values by gender (i.e., Ndofirepi, 2019; *d* = 0.11). Schwartz and Rubel (2005), using Schwartz’s original value scale, found that men tended to endorse self-enhancement values more than women, but the meta-analytic effect sizes were small (*d* = 0.20 to 0.32), and the overlap between men and women considerable.

Moving on to the age hypothesis, younger people are thought to be more concerned about environmental issues than older people, partly because environmental issues have been prominent throughout young people’s lives (Grendstad and Wollebaek, 1998), and partly because the former are less integrated into the existing social order (Van Liere and Dunlap, 1980; Grendstad and Sundback, 2003).

The evidence of the relationship between environmental concern and age, however, seems to be mixed. Although some research shows, with weak to moderate effect sizes, that younger people have stronger environmental concerns than older people (e.g., Buttel and Flinn, 1978, *r* = −0.30; Howell and Laska, 1992; Jones and Dunlap, 1992; Li and Chen, 2018, *b* = −0.03; Nord et al., 1998, β = −0.42; Vaske et al., 2011), other researchers either found no significant relationship between age and environmental concern (e.g., Gray et al., 2019), or instead found that age was positively, although weakly, related to environmental concern (e.g., Echavarren, 2017, β = 0.01; Hirsh, 2010, β = 0.13; Huddart-Kennedy et al., 2015, β= −0.03 to 0.12; Lewis et al., 2019; β= −0.40 to 0.50). There is also evidence from a large-scale, multi-country analysis, that the relationship between age and environmental concern may be weakening compared to earlier decades, at least in affluent countries (VanHeuvelen and Summers, 2019), perhaps due to increased exposure to environmental issues in the media (Howell and Laska, 1992), a finding that aligns with research of Americans from 1966 to 2009 showing that young people are becoming more concerned with money and status, and less concerned about the environment than previous generations were (Twenge et al., 2012, with moderate to large effect sizes). Therefore, the evidence in relation to the age hypothesis seems to be weak and mixed.

As the empirical relation between environmental concern and age is ambiguous at best, we would assume that age also might not be a strong predictor of environmental values, given that altruistic values are strongly related to environmental concerns (Steg et al., 2011). However, as for environmental concern, we might expect age to be negatively related to altruistic values. Indeed, Strauss et al. (2008) found a negative correlation between age and altruistic values, but the relationship was weak and non-significant (*r* = −0.12). Contradictorily, using Schwartz’s Portrait Value Questionnaire, a large multi-country study showed that people increasingly endorsed self-transcendent values more strongly, and self-enhancement values less strongly, from late adolescence to young adulthood, but then the strength of these values stabilized such that further age effects were weak (Dobewall et al., 2017).

Similar to altruistic values, we would expect the age hypothesis to predict a negative relationship between age and biospheric values – indicating that younger people more strongly endorse biospheric values. However, a weak but significant positive correlation was reported by Bonnes et al. (2011); (*r* = 0.09), suggesting, instead, an increase in biospheric values with increasing age.

Perhaps unexpectedly, the predicted relationship between age and egoistic values is also negative – younger people are predicted to have stronger egoistic values because they hold views that promote individual freedom and opportunity (Grendstad and Sundback, 2003). Strauss et al. (2008), however, found no correlation (*r* = 0.00) between age and egoistic values.

Overall, there have been very few studies where relationships between values and age have been reported, and these studies show weak and contradictory results (Strauss et al., 2008; Bonnes et al., 2011). Therefore, the conclusion in relation to the age hypothesis and altruistic, biospheric, and egoistic values remains inconclusive.

The social-class hypothesis described by Van Liere and Dunlap (1980) assumes that two specific sociodemographics are positively related to environmental concern, that is, income and educational level. In relation to the relationship between income and environmental concern, one argument is that once people have satisfied their basic physical and material needs, they will focus on higher concerns, such as the environment, which has typically been suggested by theories of need hierarchies (e.g., Maslow, 1970; Ormel et al., 1999). Empirically, environmental concern is sometimes found to increase with increasing income (e.g., Li and Chen, 2018; *b* = 0.86), and sometimes to decrease with increasing income (e.g., Dutcher et al., 2007, β = −0.12; Hirsh, 2010, β = −0.06; Huddart-Kennedy et al., 2015, β = −0.15), and is sometimes not related to income at all (Nord et al., 1998, β = 0.01). So, the evidence for the age hypothesis in relation to environmental concern seems to be contradictory.

In relation to educational levels and environmental concern, being more highly educated is presumed to enable people to become better informed about environmental and social issues (Li and Chen, 2018). There is some empirical support that educated people show more environmental concern than people with lower education levels (e.g., Buttel and Flinn, 1978, *r* = 0.23; Nord et al., 1998, β = 0.10, Van Liere and Dunlap, 1981, *r*s = 0.1 to 0.18). However, other researchers have found no relationship between educational level and environmental concern (e.g., Dutcher et al., 2007, β = 0.01; Huddart-Kennedy et al., 2015, *r* = −0.03). Some others have reported an inconsistent relationship, for example, in one study higher education was only associated with higher levels of environmental concern in a quarter of the 36 countries studied (Lewis et al., 2019).

Given the contradictory results with respect to environmental concern and social class, it is likely that the relationship between social class and environmental values will be similarly weak and inconsistent. The hypotheses with respect to social class and environmental concern could be interpreted with respect to environmental values to assume that people with higher incomes and educational levels will focus less on the self, leading to stronger altruistic and biospheric values and weaker egoistic values. Conversely, egoistic values would be more strongly endorsed by people who are less educated or less able to fulfill their basic needs.

We were unable to find any research on the relationship between income and education and environmental values. Yet, Kilbourne and LaForge (2010) found no relationship between either income or education and the importance respondents placed on material success, which could be related to egoistic values. Thus, the question of whether it is useful to segment the market by social class to target differences in environmental values is unanswered.

The residence hypothesis states that people living in urban environments are more environmentally concerned than people living in rural areas (Van Liere and Dunlap, 1980). One main reason suggested for the residence hypothesis lies in the potential higher direct exposure and information on environmental degradation, such as traffic congestion, noise, a lack of green space, and air pollution in cities (cf. Brechin and Kempton, 1994). These exposures can have various direct negative personal consequences, including health-related problems, annoyance, and diminished cognitive functioning (see, e.g., Bilotta and Evans, 2019), and, thus, urban residents, who are more likely to be affected by these negative consequences could be more concerned about the environment. Another reason for the rural-urban difference in environmental concern is the extractive-commodity hypothesis, whereby rural residents are presumed to depend more heavily on the environment for their livelihood, and therefore report less concern about the environment (Jones et al., 1999).

Some researchers investigating the relationship between residence and environmental concern have found empirical support for the residence hypothesis, whereby urban residents showed greater environmental concern than rural residents (e.g., Li and Chen, 2018, *b* = −0.17); or that size of the place of residence is positively related to environmental concern (Buttel and Flinn, 1978, *r* = 0.14). Others find very small differences (Huddart-Kennedy et al., 2009, effect sizes not reported) or no urban-rural differences in environmental concern (e.g., Huddart-Kennedy et al., 2015, β = −0.01; Nord et al., 1998, β = −0.02), or find the predicted relationship for only a few items in a larger scale (Berenguer et al., 2005). Gifford and Nilsson (2014) agree that research has yielded conflicting results, and Huddart-Kennedy et al. (2009) suggest that the difference between rural and urban residents in terms of environmental concern may be diminishing.

In terms of what research has reported with regard to the relationship between environmental concern and rural-urban residence, we would expect urban residents to more strongly endorse altruistic values, which are closely related to environmental concern (Steg et al., 2011), than rural residents. The sparse studies reporting such a relationship have shown a small difference in altruistic values in the hypothesized direction (Huddart-Kennedy et al., 2009, effect size not reported, *t* < 2).

Similarly, if urban residents are more concerned about the environment than rural residents, we would expect that relationship to hold for biospheric values. However, we are unaware of any study reporting on the relationship between biospheric values and rural versus urban residence.

It is not clear what relationship might exist between the urbanization level of the residence and egoistic values. However, Huddart-Kennedy et al. (2009) found no significant difference between the egoistic values reported by rural and urban residents (effect size not reported).

On the basis of this sparse and weak evidence in relation to environmental values and urbanization level, we expect that there will be little relationship between these variables and that, therefore, urbanization level will not be a relevant variable for market segmentation for those interesting in targeting environmental values.

According to last of the hypotheses described by Van Liere and Dunlap (1980) – the political-orientation hypothesis – people who view themselves as more left-wing (i.e., Democrats or Liberals) are assumed to be more environmentally concerned than those who view themselves as right-wing (i.e., Conservatives or Republicans). It is assumed that left-wingers are more comfortable with policies to reduce environmental problems and emphasize the negative consequences of industrial capitalism, while right-wingers show strong associations with “polluting” business and industry and defend this economic system (Dunlap, 1975).

Empirical research provides fairly clear support for the political-orientation hypothesis, as shown in a recent meta-analysis where 71 of 75 effects were in the expected direction, with a medium effect size, whereby political orientation (right – left) was significantly positively related to environmental concern (mean correlation *r* = 0.27; Cruz, 2017). Given the strength of the relationship, using political orientation profiling to target environmentally conscious consumers might be an effective strategy.

In translating the findings from environmental concern to environmental values, those who strongly endorse altruistic values usually invest more to improve the well-being of others, thus, the goal of social equality is shared by people who are both altruistic and have a left-wing orientation. As altruists strive to lessen social inequality (Thorisdottir et al., 2007), we would expect left-wingers to more strongly endorse altruistic values. Although research on the relationship between altruistic values and political orientation is sparse, negative relationships are generally found (Harring et al., 2017, *r* = −0.27; Piurko et al., 2011; *cov* = −0.11 to −0.31 in liberal countries), indicating that people who place themselves on the political left report stronger altruistic values than those on the right. Left-wingers were found to score higher on self-transcendent values (universalism: *r* = 0.28; benevolence: *r* = 0.18) than right-wingers using Schwartz’s original value scale (Caprara et al., 2006).

Given that left-wing political parties are known to support pro-environmental policies (Carter, 2013), and given that caring for others is related to environmental concern (Steg et al., 2011), we would also expect people with a left-wing orientation to report stronger biospheric values. In the only study known to us to investigate this relationship, this expectation was supported (Harring et al., 2017, *r* = −0.15), although the relationship was weaker than the same authors reported having found between political orientation and altruistic values.

Lastly, we would expect the relationship between egoistic values and political orientation to be in the opposite direction to the relationships with altruistic and biospheric values, in that we would expect people who strongly endorse egoistic values to adopt a more right-wing orientation. We expect this relationship because people who prioritize self-enhancement values should prefer non-egalitarian policies that minimize government interference in citizens’ pursuit of individual wealth and power (Piurko et al., 2011). Such a relationship was found in a study of liberal, traditional, and post-communist countries, where all reported covariances were positive (Piurko et al., 2011, *cov* = 0.10 to 0.45). Using Schwartz’s original 10-value scale, right-wing voters were found to more strongly endorse the self-enhancement values of power (*r* = −0.14) and achievement (*r* = −0.08; Caprara et al., 2006) than left-wing voters.

Given that the link between political orientation and environmental concern is medium and stable, and preliminary investigations of the relationships between political orientation and environmental values appears to be similarly convincing, political orientation may be a useful basis on which to segment the market with respect to environmental issues. We aimed to further investigate the link between political orientation and environmental values to determine whether the relationship holds for the seven countries in our sample.

Although Van Liere and Dunlap (1980) proposed the five hypotheses on how sociodemographics may be related to environmental concerns nearly four decades ago, empirical evidence for these hypotheses is mixed. Relationships between sociodemographic variables and environmental concern have been, in different studies, positive, negative, and absent. It seems likely that the evidence for the relationship between sociodemographic variables and environmental values, which have been found to be better predictors of personal norms, policy acceptability, and pro-environmental behavioral intentions than environmental concern (Steg et al., 2011), are similarly mixed. The relationship between sociodemographic variables and environmental values is of great importance because using sociodemographic variables as a market segmentation strategy might be ineffective at best, or even potentially counter effective, if the relationship is opposite to that expected.

We aimed to examine the extent to which sociodemographic characteristics are related to environmental values. We tested all five hypotheses that we based on those proposed by Van Liere and Dunlap (1980) in relation to the three environmental values in large representative samples in seven European countries; France, Greece, Hungary, the Netherlands, Norway, Switzerland, and the UK. As mentioned by other researchers (e.g., Chan et al., 2019), more cross-country research is needed as relationships can differ across countries (Chan et al., 2019; Poortinga et al., 2019).

## Materials and Methods

### Participants

Two separate samples (aiming for 1,100 participants per questionnaire per country) from France, Greece, Hungary, the Netherlands, Norway, Switzerland, and the UK were drawn from a panel by Advanced Market Research. All data were collected in 2009. In both studies, participants were selected on the basis of a number of stratification criteria (i.e., gender, age, education level, household income, marital status, and household composition) to achieve a relatively representative sample of the population for the seven countries.

Participants could only participate in one questionnaire. A total of 7,703 participants completed the first questionnaire, and 6,603 the second. We removed participants who showed irregular answering patterns from the data sets. We removed data for participants who answered more than 2/3 of all questions from one question battery identically and who filled out improbable answers in quality control questions in which we asked participants to respond to a question with a number we specified, resulting in a final sample of 6,045 and 5,775 in the final analysis. Included data did not differ substantially from the data of those who were excluded in terms of sociodemographic variables. We therefore assumed that the removal of the participants did not affect the results of this study. Our final sample was 11,820 participants; further information on the sample is in Table 1.

**TABLE 1:** Descriptive statistics for the sociodemographics.

		**Overall**	**France**	**Greece**	**Hungary**	**NL**	**Norway**	**SW**	**UK**
		
	***n***	**11,820**	**1667**	**1752**	**1696**	**1508**	**1708**	**1853**	**1636**
*Male (%)*		49.0	49.1	49.5	48.6	48.1	49.2	48.8	49.3
*Female (%)*		51.0	50.9	50.5	51.4	51.9	50.8	51.2	50.7
*Age (mean)*		43.7	44.7	40.6	41.6	45.5	42.9	43.0	47.7
**Educational level (%)**									
	*1*	3.3	6.7	2.9	1.8	2.8	3.7	3.7	1.2
	*2*	19.9	16.1	30.8	20.5	20.9	18.6	11.5	21.1
	*3*	20.8	18.4	16.6	39.3	17.9	19.8	12.5	21.8
	*4*	26.4	27.8	22.8	16.5	26.6	23.0	43.0	23.4
	*5*	29.7	31.0	26.9	21.9	31.8	34.8	29.3	32.5
**Income level (%)**									
	*% < 1,000*	23.4	14.0	18.2	81.1	17.2	5.6	7.5	21.3
	*% 1,000–2,000*	23.2	26.3	39.2	15.7	37.3	11.1	8.6	27.0
	*% 2,000–3,000*	19.7	26.8	24.5	1.4	26.5	21.0	15.1	23.7
	*% 3,000–4,000*	14.7	15.3	11.4	0.9	12.4	23.1	24.4	14.1
	*% 4,000–5,000*	9.8	12.2	2.6	0.2	3.7	15.7	24.5	8.1
	*% > 5,000*	9.2	5.5	4.2	0.6	2.9	23.5	19.9	5.9
**Urbanization level of residence (%)**									
	*1*	29.6	30.4	13.1	23.9	33.2	33.6	45.5	27.1
	*2*	40.2	41.6	38.5	38.0	42.1	38.4	40.4	42.7
	*3*	30.2	28.1	48.4	38.1	24.7	28.0	14.1	30.2
**Political orientation**									
	*Mean*	5.3	5.2	5.1	5.5	5.3	5.6	5.3	5.5

*NL, The Netherlands; SW, Switzerland.*

### Questionnaire

The two online questionnaires were part of an EU study aiming to identify barriers in household energy use. Both questionnaires included the same value scale, administered at the beginning of both questionnaires. Values were assessed with a short version of Schwartz’s (1992) value survey developed by De Groot and Steg (2008, 2010). The value scale has 13 items, including four altruistic (i.e., helpful, equality, a world at peace, and social justice), four biospheric (i.e., protecting the environment, respecting the earth, preventing pollution, and unity with nature), and five egoistic (i.e., wealth, authority, ambition, status, and social power) items. Following Schwartz, respondents indicated to what extent each value was important ‘as a guiding principle in their lives’ on a nine-point scale ranging from −1 ‘opposed to my values,’ 0 ‘not important,’ to 7 ‘extremely important.’ Respondents were urged to vary scores as much as possible and to rate no more than two values as extremely important. For this study, we focused on the items related to altruistic, biospheric, and egoistic values for which we calculated a total score as the mean of the items in each value subscale (Altruistic: *M* = 4.97, 95% CI [4.96, 4.99], Cronbach’s α = 0.75; Biospheric: *M* = 4.75, 95% CI [4.73, 4.76], α = 0.89; Egoistic: *M* = 3.05, 95% CI [3.05, 3.06], α = 0.78).

Sociodemographic items were included at the end of the questionnaires. We included the six sociodemographic variables proposed in the five hypotheses of Van Liere and Dunlap (1980) on relationships between values and sociodemographics. The sociodemographics were gender (male/female), age (years), education (1 = No education/primary school, 2 = Secondary school, 3 = High school, 4 = Vocational education, 5 = University), household income level after tax (<500, 500–1000, 1000–1500, 1500–2000, 2000–2500, 2500–3000, 3000–3500, 3500–4000, 4000–4500, 4500–5000, >5000 euros per month after tax), level of residential urbanization (1 = rural area of village, 2 = small or middle-sized town, 3 = large town), and political preference (on a scale from 1 = left, 5/6 = centre, and 10 = right wing). The descriptive information for the six sociodemographic variables for each country is presented in Table 1.

### Analyses

To test the relationship between the six sociodemographic indicators and values, we conducted multiple regression analyses to examine whether the sociodemographics predict altruistic, biospheric, and egoistic values. We used a Bonferonni correction to control for the overall Type I error rate for three regression analyses (Field, 2018). Consequently, our criterion of significance was 0.017 (0.05/3). To provide an indication for the robustness of the results, we conducted these analyses for the overall sample as well as for the samples for each country separately.

## Results

**FIGURE 1:**
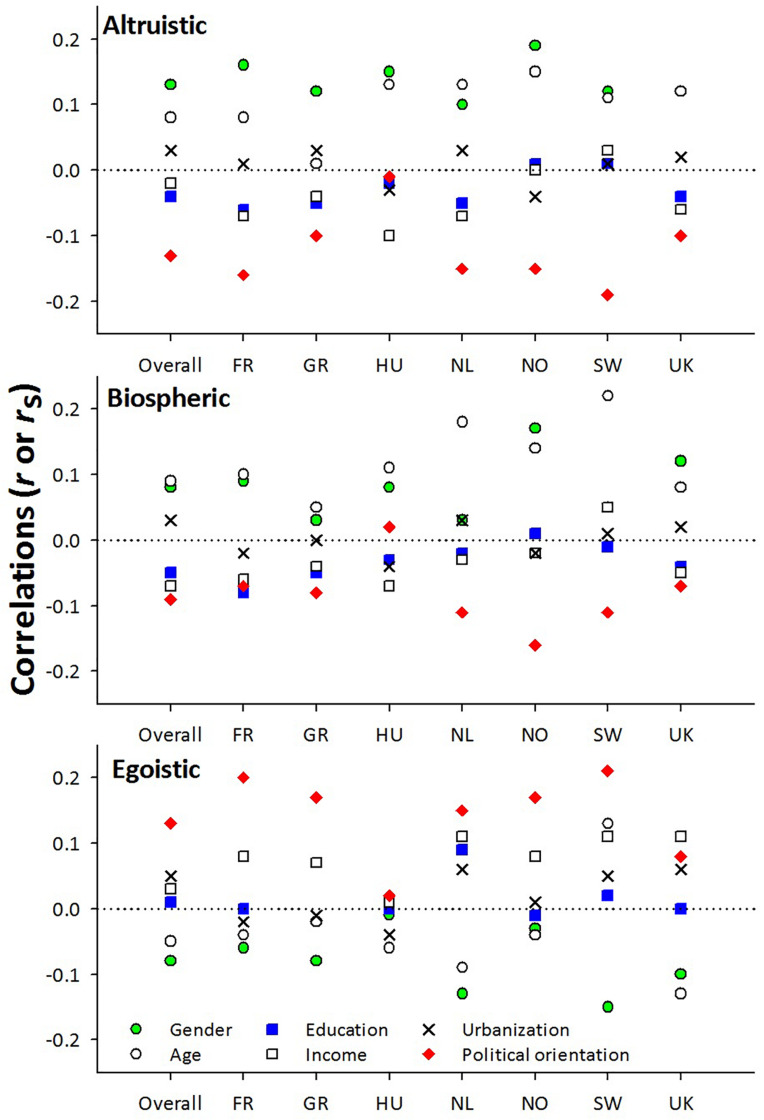
Spearman’s or Pearson’s correlation coefficients between gender, age, education, income, subjective urbanization, and political orientation and altruistic, biospheric, and egoistic values.

Figure 1 shows the correlations between the sociodemographic variables and altruistic, biospheric, and egoistic values for the overall sample and for the seven countries separately.

Gender was generally positively related to altruistic values, with correlation coefficients between 0.10 and 0.19 (all countries = 0.13). Gender was also positively related to biospheric values (*r*s between 0.03 and 0.17; all countries = 0.08), but was negatively related to egoistic values (*r*s from −0.01 to −0.13; all countries = −0.08). This indicates that male respondents scored altruistic and biospheric values as less important, and egoistic traits as more important, than women did. However, in all cases, the correlation coefficients represented small effects (Cohen, 1992).

Age was somewhat positively related to altruistic (*r*s from 0.01 to 0.15; all countries = 0.08) and biospheric (*r*s from 0.05 to 0.22; all countries = 0.09) values, but weakly negatively related to egoistic values (*r*s from −0.02 to 0.13; all countries = −0.05), indicating that older respondents scored lower on egoistic and higher on altruistic and biospheric values. Again, the *r* values indicated only weak effect sizes (Cohen, 1992).

The correlations between all three values and education, income, and subjective urbanization were very weak and inconsistent, with most individual country correlations falling below an absolute *r* value of 0.10.

Political orientation was negatively related to altruistic (*r*s from −0.10 to −0.19, all countries = −0.13) and biospheric values (*r*s from 0.02 to −0.16, all countries = −0.09), and positively related to egoistic values (*r*s from 0.02 to 0.21, all countries = 0.13). Generally, participants who rated themselves as more right wing scored altruistic aspects as less important, and egoistic aspects as more important, to them than those who were more left wing. Right-wing respondents were also less likely to score highly on biospheric values, but the correlations were weaker. Although the relationships were more consistent, they still tended to reflect small effect sizes (Cohen, 1992).

Next, we ran three linear regressions, one for each of the values, for the overall sample, and for each country separately (Table 2).

**TABLE 2:** Multiple regression analyses of sociodemographics on values in the seven sub samples.

	**France**		**Greece**		**Hungary**		**NL**		**Norway**		**SW**		**UK**	
	
	***R*^2^**	***B***	***R*^2^**	**β**	***R*^2^**	**β**	***R*^2^**	**β**	***R*^2^**	**β**	***R*^2^**	**β**	***R*^2^**	**β**
**DV: Altruistic**	**0.07**		**0.03**		**0.05**		**0.07**		**0.10**		**0.06**		**0.05**	
Gender		**0.16**		**0.12**		**0.16**		**0.13**		**0.23**		**0.12**		**0.14**
Age		**0.12**		0.02		**0.16**		**0.16**		**0.21**		**0.12**		**0.15**
Education		−0.04		−0.03		−0.03		−0.02		−0.01		−0.01		0.00
Income		−0.02		−0.02		−0.04		−0.04		0.01		0.02		−0.01
Urbanization		0.04		0.03		−0.03		0.03		−0.02		0.01		0.02
Political orientation		−**0.16**		−**0.11**		0.01		−**0.16**		−**0.16**		−**0.19**		−**0.11**
**DV: Biospheric**	**0.03**		**0.01**		**0.03**		**0.06**		**0.09**		**0.07**		**0.03**	
Gender		**0.09**		0.04		**0.08**		**0.07**		**0.20**		**0.07**		**0.13**
Age		**0.12**		0.06		**0.13**		**0.20**		**0.20**		**0.23**		**0.10**
Education		−0.05		−0.03		0.04		−0.00		0.00		−0.03		−0.02
Income		−0.04		−0.03		−0.03		−0.02		−0.01		0.03		−0.00
Urbanization		0.00		0.01		−0.03		0.04		0.00		0.01		0.03
Political orientation		−**0.07**		−**0.08**		0.05		−**0.12**		−**0.16**		−**0.11**		−**0.07**
**DV: Egoistic**	**0.05**		**0.05**		0.01		**0.06**		**0.04**		**0.09**		**0.05**	
Gender		−0.02		−**0.08**		−0.00		−**0.12**		−0.03		−**0.12**		−**0.09**
Age		−**0.09**		−0.04		−**0.07**		−**0.12**		−**0.07**		**0.09**		−**0.14**
Education		−0.04		0.05		0.02		0.03		−0.01		−0.03		−0.05
Income		**0.08**		**0.07**		0.03		**0.09**		**0.07**		**0.09**		**0.08**
Urbanization		−0.02		−0.00		−0.04		0.06		0.00		**0.06**		**0.06**
Political orientation		**0.20**		**0.17**		0.02		**0.13**		**0.17**		**0.19**		**0.10**

*DV, dependent variable; NL, The Netherlands; SW, Switzerland. Numbers in bold are significant at *p* < 0.017 (Bonferonni correction).*

### Altruistic Values

The six sociodemographic variables explained 5% of variance in altruistic values, *F*(6,11813) = 102.50, *p* < 0.001, in the overall sample. All sociodemographic variables, except income level, were significant predictors (with *p* < 0.001) of altruistic values (gender, *b* = 0.36, β = 0.15; age, *b* = 0.01, β = 0.11; education, *b* = −0.03, β = −0.03, income, *b* = 0.003, β = 0.01, *p* = 0.43; urbanization level, *b* = 0.06, β = 0.04; political orientation, *b* = −0.07, 0.36, β = 0.13). People who were older, left-wing, female, and lived in more urban areas were more likely to value altruistic aspects in life compared to those who were younger, right-wing, male, and rural dwellers. Looking at the countries separately, sociodemographics explained between 3% (Greece) and 10% (Norway) of the variance (Table 2) in altruistic values. Gender and age positively and uniquely contributed to the model in all seven countries (but age was not a significant predictor for Greece), while political orientation was negatively related to altruistic values (but not significantly for Hungary). Education, income, and subjective urbanization did not contribute significantly to altruistic values when controlling for other predictors. Overall, the sociodemographic variables measured did not predict much of the variance in values across our samples, or overall, and the regression coefficients were small.

### Biospheric Values

The six sociodemographic variables explained 3% of the variance in biospheric values, *F*(6,11813) = 60.01, *p* < 0.001, in the overall sample. All sociodemographic variables were significant predictors (with *p* < 0.001) of biospheric values, although the standardized and unstandardized regression coefficients were generally lower than for altruistic values (gender, *b* = 0.24, β = 0.08; age, *b* = 0.01, β = 0.11; education, *b* = −0.04, β = −0.04, income, *b* = −0.02, β = −0.04; urbanization level, *b* = 0.06, β = 0.03; political orientation, *b* = −0.06, β = −0.09). People who were older, left-wing, and female endorsed biospheric values more strongly than those who were younger, right-wing, and male. People with lower incomes, educational level, and those living in urban areas showed stronger endorsements of biospheric values than those with higher incomes, educational level, and living in rural areas. Sociodemographics explained between 1% (Greece) and 9% (Norway) in the variance in biospheric values in the seven country samples (Table 2). Gender and age generally contributed positively and uniquely to the explanation of biospheric values across the seven countries (except for Greece), while political preference seemed to contribute negatively to these values (except for Hungary). Education, income, and subjective urbanization did not contribute significantly to biospheric values when controlling for other predictors. As with altruistic values, the regression models did not account for much of the variance in biospheric values, and the regression coefficients were low.

### Egoistic Values

In the overall sample, the six sociodemographic variables explained 3% of the variance in egoistic values, *F*(6,11813) = 66.34, *p* < 0.001. All sociodemographic variables, except education level, were significant predictors (with *p* < 0.001) of egoistic values (gender, *b* = −0.20, β = −0.08, age, *b* = −0.01, β = −0.07; education, *b* = −0.01, β = −0.01, *p* = 0.29; income, *b* = 0.02, β = 0.04; urbanization level, *b* = 0.09, β = 0.05; political orientation, *b* = 0.08, β = 0.14). Participants who were right-wing, male, young, lived in urban areas, and had higher incomes more strongly endorsed egoistic values than people who were left-wing, female, older, lived in rural areas, and had lower incomes. Regression models were similar for the seven countries, with explained variances ranging from 0.01 to 0.09 (see Table 2). The six sociodemographic variables did not significantly predict egoistic values in the Hungarian sample; the model was not significant (*R*^2^ = 0.01). The unique contribution of the six predictors was somewhat different across the remaining six countries, with political preference and income being stable significant predictors across samples. Gender only contributed significantly to the model in Greece, the Netherlands, Switzerland, and the UK. Although age contributed significantly to the explanation of egoistic values in six of the seven countries (but not in Greece), interestingly, the direction of the relationship was negative for the Swiss sample. Urbanization level only contributed uniquely to the explanation of the variance in egoistic values in the Swiss and UK sample, but education was not a significant predictor in any country. The overall variance accounted for in egoistic values by sociodemographic variables was low, and regression coefficients were small.

## Discussion

Sociodemographics provide a popular basis for profiling or segmenting a population, and could be useful for tailoring campaigns or messages with environmentally friendly motives. However, results regarding the relationships between environmental values and sociodemographic variables are rare, mixed, and inconclusive. We aimed to investigate the extent to which sociodemographic variables predict environmental values in line with the five hypotheses as proposed by Van Liere and Dunlap (1980). We further validated these relationships by using large representative samples in seven European countries. Overall, for all three environmental values, the regression models accounted for very little of the variance in the model, suggesting that sociodemographic variables are not particularly predictive of these values overall. There are clearly other variables accounting for the variability in environmental values – and these other variables may prove to be more promising for market segmentation than sociodemographic variables. For example, Schwartz and Rubel (2005) suggested that the importance of different values may vary with culture, and, additionally, that culture may moderate the relationship between demographic variables (such as sex) and value orientations. Future researchers should examine cultural differences in environmental values with a wider number of countries than we used here.

Although all relationships between the sociodemographic variables and environmental values were weak, at best, women endorsed altruistic, and to a lesser extent, biospheric values, slightly more strongly than men, and men endorsed egoistic values slightly more strongly than women. This pattern was fairly consistent across the seven countries, and similar to the patterns generally found by other researchers for environmental concern or values (e.g., Mohai, 1992; Blocker and Eckberg, 1997; Zeleny et al., 2000; Vaske et al., 2001; Kalof et al., 2002; Casey and Scott, 2006; Dutcher et al., 2007; Swami et al., 2010; Burn et al., 2012; Huddart-Kennedy et al., 2015; Xiao and McCright, 2015; Milfont and Sibley, 2016; Echavarren, 2017; Mueller and Mullenbach, 2018).

Age also emerged as a weak predictor of values, but, unexpectedly, some relationships were opposite to those generally found. That is, as expected (according to Grendstad and Sundback, 2003, among others), older respondents were slightly less likely to endorse egoistic values, but, unexpectedly, slightly *more* likely to endorse altruistic and biospheric values than younger respondents (in line with the results of Bonnes et al., 2011). These findings perhaps support the contention that the relationship between age and biospheric concern is weakening (Howell and Laska, 1992; Twenge et al., 2012). However, past research has often produced weak or contradictory results and it may be that the relationship between age and biospheric values is not stable across contexts.

Political preference was the last predictor that was slightly more strongly related to values for our samples. In line with previous research, left-wing respondents endorsed altruistic (Piurko et al., 2011) and biospheric values (Harring et al., 2017) slightly more than right-wing respondents, who endorsed egoistic values slightly more strongly (Choma et al., 2010; Piurko et al., 2011; Harring et al., 2017). These relationships were consistent across countries, and significant in all countries, except Hungary. In post-communist countries, such as Hungary, the labels “left” and “right” may have less meaning as citizens of these countries have not yet developed a common understanding of the left–right dimension (Piurko et al., 2011). Therefore, as Piurko et al. (2011) found, no clear relationship with values and political orientation emerges in post-communist countries. However, for the other six countries, the finding that political orientation was consistently related to values that are important determinants of environmental behavior suggests that political orientation may be a relevant, although fairly weak, sociodemographic variable on which to base a segmentation strategy.

Income, education, and subjective urbanization were essentially unrelated to altruistic or biospheric values (Figure 1 and Table 2), therefore our results did not align with those showing support for the social-class (Buttel and Flinn, 1978; Van Liere and Dunlap, 1980, 1981; Arcury and Christianson, 1990; Nord et al., 1998; Bodur and Sarigöllü, 2005; Casey and Scott, 2006; Li and Chen, 2018), or residence hypotheses (Arcury and Christianson, 1990; Jones and Dunlap, 1992; Li and Chen, 2018). As our large sample sizes provided adequate power to detect relationships, if they had existed, we believe that income, education, and subjective urbanization are not likely to provide effective strategies for market segmentation strategies related to altruistic or biospheric values.

Counter to expectations, income was significantly, although weakly, *positively* related to egoistic values in six of the seven countries (not in Hungary), whereby those with higher incomes were *more* likely than those with lower incomes to rate egoistic values as important to them. Incomes in Hungary were generally very low compared to the other countries, meaning that there was less variability in income using our income scale. Due to the fact that the scale was ordinal, it was not possible to re-scale the variable in terms of relative income, which Li and Chen (2018) showed was a stronger predictor of environmental concern than absolute income.

Subjective urbanization was only significantly (although weakly) predictive of egoistic values in Switzerland and the UK, where a greater degree of urbanization was predictive of stronger egoistic values. It is not clear why subjective urbanization would be particularly related to egoistic values in these two countries, and we tentatively suggest that the relationships are of marginal importance, given their small effect sizes. Additionally, although the relationship between subjective urbanization and egoistic values was positive for Switzerland and the UK, it was negative (but non-significant) for France and Hungary, suggesting that any relationship between the variables is weak and inconsistent, and therefore unlikely to be useful for segmentation strategies. Lastly, education was not significantly predictive of egoistic values in any country, suggesting that education is also of little interest.

### Limitations and Directions for Future Research

We used commercial opt-in panels to collect our data set. Some claim that data collected in this way may differ from data collected using more traditional data collection methods (Yeager et al., 2011; Hays et al., 2015). However, recent meta-analytic evidence suggests that convenience samples, like those obtained using opt-in panels, are appropriate when researchers are seeking to understand relationships between variables, rather than to generalize point estimates, such as mean values, to a population (Walter et al., 2019). Our samples were representative of each country’s population in that we selected participants on the basis of a number of stratification criteria (i.e., gender, age, education level, household income, marital status, and household composition). Our results suggest, however, that achieving such representative samples may not be necessary when investigating environmental values, given that sociodemographics were not very predictive of such values.

## Conclusion

Overall, we found little evidence that the sociodemographic variables of gender, age, social class, urbanization of residence, and political orientation are useful predictors of environmental values. All sociodemographics were weakly related to environmental values, and we conclude that there are likely other variables that would better predict values related to the environment.

Our findings may be relevant to policymakers, marketers, and those wishing to design tailored environmental advertising, policies, and campaigns using market segmentation approaches. In the absence of information about the environmental or social values of a populace, it may be appropriate to infer, on a general, population level, that right-wing, younger men are the least likely to value environmental or altruistic policies. Policies designed to produce pro-environmental outcomes might be best couched in economic terms to gain the support of these citizens. The evidence for the relationships between environmental values and both social class and residential location is weak and the relationship with age is equivocal. There are other variables that predict environmental behavior, such as attitudes and personal and social norms (Klöckner, 2013) and these variables may be more strongly related to sociodemographic variables than environmental values. Until such relationships are established by future researchers, however, campaigns targeted at sociodemographic segments may be no more effective than general campaigns, and, at worst, they may produce effects opposite to those intended.

## Data Availability Statement

The raw data supporting the conclusions of this article will be made available by the authors, without undue reservation.

## Ethics Statement

Ethical review and approval was not required for the study on human participants in accordance with the local legislation and institutional requirements. The patients/participants provided their written informed consent to participate in this study.

## Author Contributions

LS was involved in the study conception and data collection. JD wrote the first version of the manuscript. RS substantially revised the first draft, and analyzed the data for the final manuscript. All authors contributed to the content of the manuscript.

## Conflict of Interest

The authors declare that the research was conducted in the absence of any commercial or financial relationships that could be construed as a potential conflict of interest.
